# Editorial: Therapeutic strategies to lower residual dyslipidemic CV risk beyond LDL-C and statins

**DOI:** 10.3389/fcvm.2025.1597062

**Published:** 2025-06-27

**Authors:** Raffaele De Caterina, Matthew Budoff, William E. Boden

**Affiliations:** ^1^ Pisa University Hospital, University of Pisa, Pisa, Italy; ^2^ Preventive Cardiology, Lundquist Institute for Biomedical Innovation, Torrance, CA, United States; ^3^ VA Boston Healthcare System, Department of Veterans Affairs (VA), Professor of Medicine, Boston University School of Medicine, Senior Lecturer, Harvard Medical School, Boston, MA, United States

**Keywords:** statin, cardiovascular disease, Icosapent ethyl, Omega-3 fatty Acids, secondary prevention, primary prevention

**Editorial on the Research Topic**
Therapeutic strategies to lower residual dyslipidemic CV risk beyond LDL-C and statins

Atherosclerotic cardiovascular disease (ASCVD) remains the leading cause of morbidity and mortality worldwide (1). Cardiovascular (CV) risk reduction is important in patients at high risk for a first event (primary prevention) and those with established ASCVD (secondary prevention). For decades, statin therapy has been the main therapeutic agent to reduce the risk of CV events. However, even in patients with low-density lipoprotein cholesterol (LDL-C) levels well below guideline-recommended targets, a substantial risk for CV events remains. Therefore, methods are needed to address persistent CV risk in patients taking statins, particularly with additional agents that target pathways beyond LDL-C. Components of the residual risk include inflammation, thrombotic and metabolic factors, and elevated triglyceride (TG) levels.

This Research Topic of *Frontiers* aims at providing a forum for current advances in reducing persistent CV risk in patients taking statins and with persistently elevated TG levels, with a special focus on use of icosapent ethyl (IPE) or eicosapentaenoic acid (EPA), recently come to the worldwide attention after the strikingly favourable findings from the *Reduction of Cardiovascular Events with Icosapent Ethyl–Intervention Trial* (REDUCE-IT) (2, 3). Omega-3 fatty acids and fibrates have been studied for reducing residual CV risk. In this context, fibrates and mixed omega-3 fatty acids containing EPA and docosahexaenoic acid (DHA) have not resulted in CV benefit on top of statin therapy. However, EPA-only formulation in IPE has consistently reduced CV risk in patients taking statins who have established CV disease or in those with diabetes and other risk factors for a first CV event (3).

Currently, IPE/EPA is the only available CV prevention agent that reduces ASCVD risk by targeting pathways beyond LDL-C reduction in patients treated with statins who have persistently elevated TG levels.

This Research Topic of *Frontiers in Cardiovascular Medicine - Cardiovascular Pharmacology and Drug Discovery* – has collected a series of 7 papers including Original Research Articles, Mini Reviews and Reviews related to strategies to reduce residual cardiovascular risk beyond LDL-C lowering.

In a first introductory *Review* by Vijayaraghavan et al. the authors have reviewed the contemporary literature assessing intermediate- and long-term event rates in patients with established CV disease treated with statins. The authors show CV event rates among patients treated with statins who have established CV disease, including coronary artery disease, cerebrovascular disease, or peripheral arterial disease, accumulate over time, with a cumulative incidence of CV events reaching up to approximately 40% over 10 years. Recurrent stroke occurs in up to 19% of patients seven years after a first cerebrovascular event. Repeat revascularization and CV-related death occur in up to 38% and 33% of patients with peripheral artery disease after three years, respectively. Thus, it appears, with compelling arguments, that additional treatment strategies are needed to reduce persistent CV risk in patients with established CV disease treated with statins.

In a second *Mini-Review* by Nelson et al. the authors have reviewed the effect of statin add-on therapy on cardiovascular mortality, highlighting findings related to the primary composite CV endpoints and the more patient-centric endpoint of CV-related mortality. Add-on therapies here examined have included ezetimibe, proprotein convertase subtilisin/kexin type 9 (PCSK9) inhibitors, bempedoic acid and IPE. Although all such treatments are associated with a reduction in CV events, only add-on therapy with IPE demonstrated a significant reduction in CV mortality in the overall intent-to-treat population, possibly due to the unique pleiotropic mechanisms of IPE/EPA, independent of lipid-lowering effects.

In the *Narrative Review* by Bashir et al., the authors focus on hypertriglyceridaemia as an important component of residual risk. On this topic, large population-based observational studies have consistently demonstrated an association between hypertriglyceridaemia with ASCVD. This relationship is complicated by the co-existence of low high-density lipoprotein cholesterol. Despite significantly improving atherogenic dyslipidaemia, the most recent clinical trial outcomes have cast doubts on the utility of pharmacologically lowering triglyceride concentrations using fibrates. On the other hand, purified EPA, but not in combination with DHA, has produced favourable ASCVD outcomes. The outcome of these trials suggests alternate pathways involved in ASCVD risk modulation, with a particular focus on still incompletely clarified components of residual risk beyond hypertriglyceridaemia itself and addressed by EPA.

In the *Brief Research Report* by Nelson et al., the authors report on a cross-sectional analysis of demographic and clinical characteristics of patients in the United States using Icosapent Ethyl. Among patients with ≥2 IPE prescriptions and triglyceride data, 28.0% had severe hypertriglyceridaemia. In the secondary prevention cohort, coronary artery disease was the most common pre-existing CV disease, and many had diabetes. Most were already receiving statin treatment. Thus, patients taking IPE had characteristics consistent with its current indication, including well-controlled LDL-C levels with statin use. The higher triglyceride levels before IPE initiation suggest that IPE may be underutilized in patients at high risk for CV events.

In the *Review* “*Global eligibility and cost effectiveness of icosapent ethyl in primary and secondary cardiovascular prevention*” by Toth et al., the authors aimed at summarizing information from eligibility and cost effectiveness studies of IPE to date. In a total of sixteen studies, involving 2,068,111 patients in the primary or secondary prevention settings worldwide, the authors found that up to 45% percent of patients were eligible for IPE, depending on the selection criteria used (i.e., REDUCE-IT criteria, US Food and Drug Administration label, Health Canada label, practice guidelines) and the population studied. Overall, findings indicated that IPE is particularly cost effective in patients with established CVD.

Two original research papers here published further broaden our understanding of the mode of action of Icosapent ethyl. In the paper by Le et al., higher levels of DHA, likely competing with EPA, were found to lower the protective impact EPA on long-term major cardiovascular events in a cohort of 987 randomly selected subjects enrolled in the INSPIRE biobank registry who underwent coronary angiography, in whom rapid throughput liquid chromatography-mass spectrometry could quantify EPA and DHA plasma levels. These results may explain the inconsistency of outcome findings with combined preparations of EPA and DHA, contrary to what reported with IPE/EPA alone in REDUCE-IT.

Finally, in the *Original Research* paper on quantitative imaging biomarkers of coronary plaque morphology by Buckler et al., with findings from the *Effect of Vascepa on Improving Coronary Atherosclerosis in People With High Triglycerides Taking Statin Therapy* (EVAPORATE) study (4), the authors further evaluated the effects of IPE on plaque characteristics by coronary computed tomography angiography. Plaque morphology, including lipid-rich necrotic core, fibrous cap thickness, and intraplaque haemorrhage, was here assessed using advanced bioimaging analyses of computed angiography images. The authors found a decrease of lipid-rich necrotic core between the patients on IPE vs placebo at 9 months, widening at 18 months, and accompanied by reductions in wall thickness and increases in cap thickness. These findings provide a mechanistic insight for the interpretation of REDUCE-IT outcomes, highlighting the acquisition of characteristics of greater atherosclerotic plaque stability in patients treated with IPE.

We believe this collection of manuscripts is a valuable addition to the body of knowledge on strategies to reduce residual risk and highlight important mechanisms for the beneficial effects of IPE/EPA in cardiovascular patients.
